# The interplay of circulating tumor DNA and chromatin modification, therapeutic resistance, and metastasis

**DOI:** 10.1186/s12943-019-0989-z

**Published:** 2019-03-09

**Authors:** Lei Zhang, Yiyi Liang, Shifu Li, Fanyuan Zeng, Yongan Meng, Ziwei Chen, Shuang Liu, Yongguang Tao, Fenglei Yu

**Affiliations:** 1 Key Laboratory of Carcinogenesis and Cancer Invasion, Ministry of Education, Department of Pathology, Xiangya Hospital, Central South University, 87 Xiangya Road, Changsha, 410008 Hunan China; 2 NHC Key Laboratory of Carcinogenesis (Central South University), Cancer Research Institute and School of Basic Medicine, Central South University, 110 Xiangya Road, Changsha, 410078 Hunan China; 3 Department of Oncology, Institute of Medical Sciences, Xiangya Hospital, Central South University, 87 Xiangya Road, Changsha, 410008 Hunan China; 4 Department of Thoracic Surgery, Second Xiangya Hospital, Central South University, Changsha, 410011 China

**Keywords:** ctDNA, Chromatin modification, Therapeutic resistance, Metastasis, Tumor heterogeneity

## Abstract

Peripheral circulating free DNA (cfDNA) is DNA that is detected in plasma or serum fluid with a cell-free status. For cancer patients, cfDNA not only originates from apoptotic cells but also from necrotic tumor cells and disseminated tumor cells that have escaped into the blood during epithelial-mesenchymal transition. Additionally, cfDNA derived from tumors, also known as circulating tumor DNA (ctDNA), carries tumor-associated genetic and epigenetic changes in cancer patients, which makes ctDNA a potential biomarker for the early diagnosis of tumors, monitory and therapeutic evaluations, and prognostic assessments, among others, for various kinds of cancer. Moreover, analyses of cfDNA chromatin modifications can reflect the heterogeneity of tumors and have potential for predicting tumor drug resistance.

## Biological features of ctDNA

**Fig. 1:**
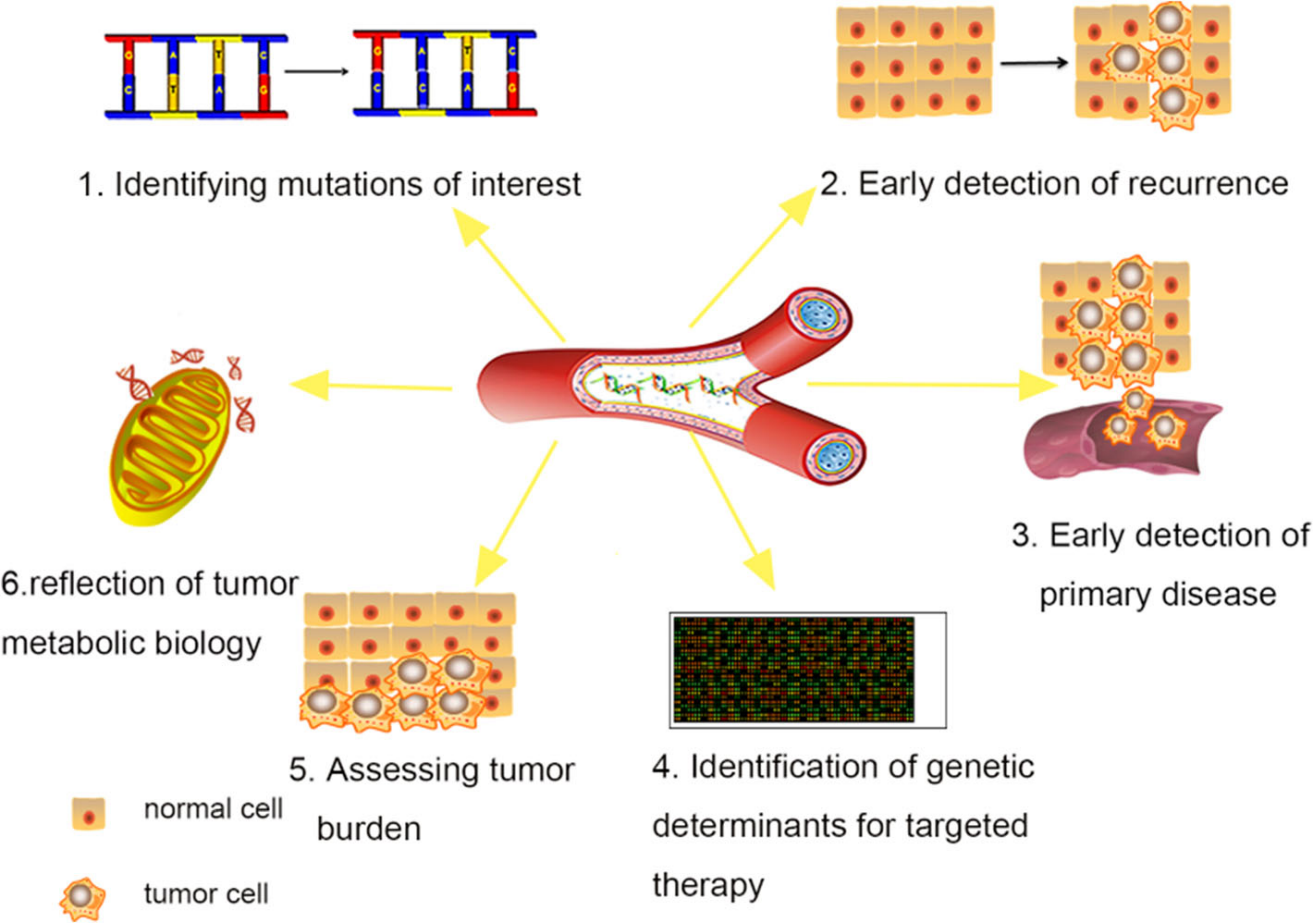
The clinic applications of ctDNA include (1) ctDNA to identify mutations of interest (including resistance mutations), (2) early detection of disease recurrence in minimal residual disease, and (3) early detection of primary disease. (4) Identification of genetic determinants for targeted therapy. (5) Serial ctDNA quantification to assess tumor burden. (6) Reflection of tumor metabolic biology

Peripheral circulating free DNA is DNA that is detected in plasma or serum fluid with a cell-free status. It may originate from apoptosis or necrosis. The amount of circulating DNA in healthy people is minimal. However, when the body is afflicted by tumors, autoimmune disease, and inflammatory reactions, the amount of circulating free DNA in the body can increase in correlation to different disease statuses [1]. Circulating free DNA is often a double-stranded DNA fragment that exists in the form of a protein complex [2]. The size of the cfDNA varies, with a length ranging from 18 bp to 10000 bp. Among cfDNAs, circulating tumor DNA (ctDNA), which is believed to come from tumor cells, has attracted significant interest from researchers. Nucleosomes released together with ctDNA, acting as carriers, enter the blood in single, double or triple forms, and thus most ctDNA shows significant fragmentary characteristics. Moreover, the half-life of ctDNA in the blood circulation is less than 2 hours [3]. Additionally, tumor-specific changes (changes in DNA integrity [4], mutations in oncogenes or tumor suppressor genes [5], gene methylation abnormalities, microsatellite alterations [6], changes in mitochondrial DNA load levels [7], chromosomal genomes rearrangements, etc.[5]) can be detected in the ctDNA of cancer patients. In summary, ctDNA is not only easy to identify, but its blood concentration can also reflect the latest developments and specificity of tumors in real time [8]. The applications of ctDNA include the followings: (1) identifying mutations of interest, including mutations responsible for resistance to therapy, in ctDNA; (2) early detection of disease recurrence in minimal residual diseases; (3) early detection of primary disease; (4) identification of genetic determinants for targeted therapy; (5) serial ctDNA quantification to assess tumor burden; (6) reflection of the metabolic biology of tumors. (Fig. 1)

## Origin and test methods for ctDNA

### Origin of ctDNA

In healthy human plasma, cfDNA mainly derives from apoptotic cells [9]. In addition, all living cells spontaneously release DNA fragments, which are called metabolic DNA fragments, into the blood. Metabolic DNA fragments have practical biological functions, such as acting as transcriptional templates of RNA and binding to glycoproteins as messengers. However, cfDNA can originate from necrotic and apoptotic tumor cells [10]; exosomes derived fromcancer cells [11, 12]; disseminated tumor cells (DTC), which may intravasate from a solid tumor and travel through the blood stream and subsequently extravasate into distant organs, such as the bone marrow [13]; and circulating tumor cells (CTCs), which are tumor cells that have, presumably, been released or passively shed from the primary tumor and/or metastatic lesions into the bloodstream [14–17] in cancer patients.

The amount of ctDNA in a patients’ blood is closely correlated with tumor burden and increases significantly with tumor growth [9]. If a patient carries a tumor weighing approximately 100 g (equivalent to 3×10^10^ cells), 3.3% of the tumor-derived DNA will be released into the blood each day given that a single human somatic cell contains approximately 6.6 pg of genomic DNA [18]. A study based on cfDNA analysis of 45 breast cancer patients, 42 colorectal cancer patients, 65 lung cancer patients, 42 ovarian cancer patients and 44 healthy people showed that cancer patients present elevated cfDNA level of 29 ng/ ml compared with 7 ng/ ml of healthy people.[19] Size, status, and characteristics of cancerous tissues are also highly related. For example, when considering intratumor heterogeneity, subclones carrying driver mutations are more prone to release DNA [20]. However, some researches [21] in non-small cell lung cancer (NSCLC) have indicated that the cfDNA level is correlated with tumor metabolism and reflects tumor biological behaviors rather than tumor burden, potentially because nontumor DNA is also increased during tumor progression due to interactions between tumor cells and adjacent healthy tissue cells [22].

**Fig. 2:**
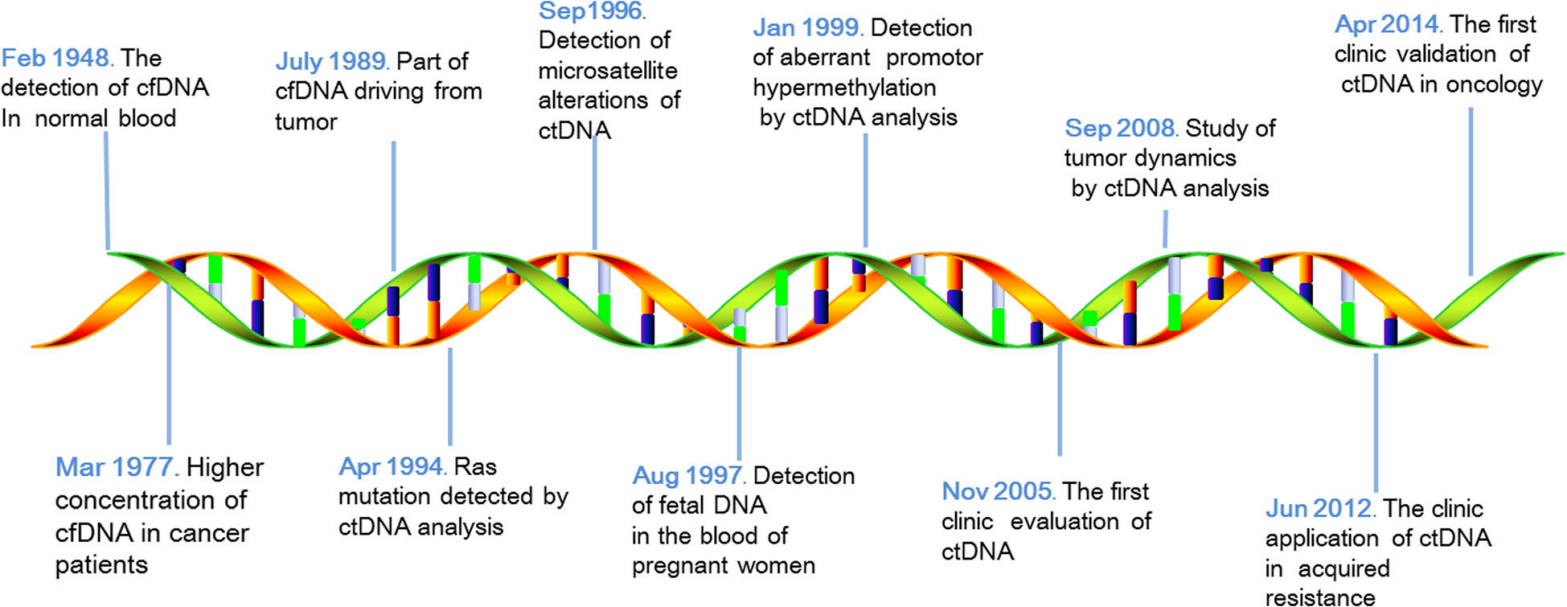
Timeline of the main important discoveries of ctDNA

The earliest attempt to analyze ctDNA, however, date to the 1950s. Mandel [23] detected floating DNA fragments (cell-free DNA, cfDNA) in normal blood in 1948; however, their first driving work did not receive sufficient attention. In 1977 [24], it was evident that the content of DNA in the blood of tumor patients was significantly higher than that in healthy individuals, especially in advanced tumor patients. However, it was not until 1989 that researchers discovered the presence of fragments of cfDNA in the plasma and serum of cancer patients with the same genetic changes as the tumor [25]. Mutated K-ras sequences were identified in plasma DNA from three patients with pancreatic carcinoma in 1994 [26], and in the same year, point mutations of the N-Ras gene were also demonstrated in the plasma DNA of patients with myelodysplastic syndrome or acute myelogenous leukemia [27]. Since then, the concept of ‘liquid biopsy’ was born. Microsatellite analysis of serum as a novel method detected the microsatellite alterations of circulating tumor cell DNA of small cell lung cancer patients [28] and head and neck cancer patients in 1996 [6]. Three years later, in 1999, aberrant promoter hypermethylation of cancer-related genes in serum was detected by methylation-specific PCR, which may be useful for cancer diagnosis or the detection of recurrence [29] (Fig. 2).

Although extensive researches had been performed, such studies remained to be validated in clinical practice. The clinical evaluation of ctDNA alterations was first proposed in 2005 [18]. Subsequently, ctDNA measurements were used to responsibly monitor the dynamics of tumor burden [30] and analyze acquired resistance to cancer treatments [31, 32]. In a comprehensive study in 2014, the clinical validation of ctDNA analysis in oncology was first proposed [33]. Moreover, DNA from serum or blood provided another clinic domain: fetus-derived Y sequences were detected in pregnant women’s blood in 1997, which indicated that fetal DNA could enter the blood [34]. Thus, a noninvasive prenatal diagnosis became possible. cfDNA researches later focused more attention on cancer research rather than prenatal diagnosis, potentially because detecting tumor DNA is much more difficult than fetal DNA due to early, inaccurate sequencing technologies.

### Test methods for ctDNA

The first step in detecting cfDNA is to extract free DNA from the peripheral blood within 4-5 hours after drawing 1 ml serum or plasma. Taking lung cancer as an example, there are many detection methods for single gene mutations (such as epidermal growth factor receptor (EGFR) mutations) in cfDNA, such as liquid chromatography, the mutation amplification block method, digital PCR, and second-generation sequencing. Overall, the BEAMing digital PCR method is the most sensitive approach, providing a sensitivity reaching 0.01% in comparison to that of other methods of approximately 1%. The four methods used to detect ctDNA EGFR mutations comprise two amplification refractory mutation systems (cobas-ARMS and ADx-ARMS), a droplet digital polymerase chain reaction (ddPCR) and next-generation sequencing (Firefly NGS) platform. Firefly NGS, cobas-ARMS and ddPCR are more sensitive than ADx-ARMS, while ADx-ARMS is suitable for the quantitative detection of EGFR mutations with an allele frequency greater than 1% [35]. However, ctDNA sequencing methods, such as NGS, still sometimes cannot reflect all the somatic mutations in biopsy tissue [36], and thus a detection approach with high sensitivity is anticipated **(**Table 1).

**Table 1:** Common ctDNA Analysis Techniques. Description of Twelve Common Methodologies of ctDNA Analysis

Technique	Main features	Description	Accuracy, sensitivity, specificity	Advantages	Challenges or perspectives	Ref.
Hybrid-capture-based Liquid Biopsy Sequencing (LB-Seq)	A hybridization-based method sequencing all protein-coding exons	Barcoded cfDNA-seq libraries design, probe hybridization, target capture, post-capture amplification and bead clean up of captured amplified DNA	AFs: 0.25% specificity: 98%	1. High fidelity 2. Screening for mutations throughout a diversity of genomic regions	Larger portions of the genome to query other target genes or mutation classes like rearrangements and copy number alterations	[39]
DNA clutch probes (DCP)	Without enzymatic amplification but a DCP used to prevents the reassociation of ssDNAs	ctDNA denaturization, DCP preventing reassociation of ssDNA, PNA clamps hybridizing to the matched wild type, detection of remaining single-stranded mutant target ctDNA	Detect 0.01% mutations	1. High specificity with less time requirement 2. Chip-based format supports automation.	Monitoring diseases caused by DNA viruses	[40]
iDES-enhanced CAPP-Seq	Combining in silico elimination of highly stereotypical background artifacts with a molecular barcoding strategy for the efficient recovery of cfDNA molecules	Designing ‘index’ barcode and‘insert’ barcodes, PCR, mapping to reference genome to recover single strand, duplex recovery, in silico reassembly of original DNA duplex	4 in 105 cfDNA molecules	Increased scalability, flexibility, coverage uniformity, and ability to reliably assess all mutation classes in a single assay	Allowing for greater analytical sensitivity than iDES if >~200 somatic mutations were targeted	[41]
Targeted error correction sequencing (TEC-Seq)	A direct evaluation of sequence changes in circulating cell-free DNA using massively parallel sequencing	including dual-index barcode adapters design, cfDNA library formation, redundant sequencing of the library, reconciliation of duplicate fragments, alignment to the reference genome, identification of bona fide alterations.	Sensitivity: 97.4% specificity>99.9999%	Sensitive and highly specific detection of low-abundance sequence alterations using NGS	Sensitivity may be further improved by deeper sequencing, improved error correction methods, larger blood volumes, and repeated testing at regular intervals.	[19]
Nanoplasmonic biosensor	Localized surface plasmon resonance (LSPR) and the coupling plasmon mode of gold nanoparticles (AuNPs) for enrichment strategy.	A change of the refractive index surrounding the biosensor surface for binding of ctDNA to the PNA-probed AuNP surface. Change of RI as distinct LSPR-peak changes on the Rayleigh light scattering. Detection and amplification of methylation by specifically binding immunogold colloids	Sensitivity: four times (~50 fM) improvement	Simultaneous detection of the hot-spot mutation and epigenetic changes on the ctDNA	Providing sharp sensitive and multiplexed platform for detecting other associated biomarkers and their modifications at low concentration.	[42]
Simple multiplexed PCR-based barcoding of DNA	Detection of extremely rare variant alleles within a complex mixture of DNA molecules	Comprising a three-cycle barcoding PCR step followed directly by adaptor PCR to generate the library and then bead purification before sequencing	Error correction to <0.1%,	1.Simplicity of the NGS library construction protocol and the ease in any reasonably capable research laboratory 2. The low DNA input (<5 ng),	1. time-consuming, and not be the best approach for coverage of consistent, large target regions on many samples. 2. Requirement of deep sequencing, and sequencing costs	[43]
Sensitive digital quantification of DNA methylation in clinical samples	Providing an opportunity to assess DNA methylation with allele-specific PCR, restriction digestion or specific hybridization probes	Digital approaches involve the counting of methylated and unmethylated fragments, one-by-one, thereby dramatically increasing the signalto-noise ratio of the assay.	the methylated DNA fraction was 0.018%	1.enabling increased sensitivity and specificity 2.enabling comparisons across different patient cohorts for standardized clinical interpretations		[44]
Nanostructured conductive polymer platform	Extracting tumor-specific circulating cfDNA from unprocessed plasma using an electroactive Ppy/Au NW platform	Ppy-coated Au nanowires (Ppy/Au NWs) capture DNA with oxidation electric fields by DNA-Ppy surface adsorption, while Ppy/Au NWs release DNA with reduction electric fields.	mean purity: 1.97 ± 0.02	Enhanced efficiency, high yield and high purity	_	[45]
Tagged-amplicon deep sequencing (TAm-Seq)	Combining short amplicons, two-step amplification, sample barcodes with high-throughput PCR	Preamplification of DNA molecules with or without mutations, single-plex PCR to select region of interest, barcoding PCR to harvest amplicons duplicate sequencing to avoid false positives caused by PCR errors	AF: 2%	1. A balance between sensitivity and ease of use 2. Effective amplification 3. Sample barcodes and high-throughput PCR 4. less time	Challenge: detection limit compared to assays that target individual loci	[46]
Single copy sensitive electrochemical Assay	Schematic representation of the SEDA strategy.	Integrated by dual sequence discrimination processes including methylation-specific annealing and specific interface hybridization, as well as cascade signal amplification processes represented by the asymmetric MSP and HRP catalytic reaction.	The high specificity reaching a 0.1% methylation index	1. Integrated by dual sequence discrimination processes and cascade signalamplification processes 2. Detection of tumor related methylation in lung cancer patients with 200 microlitre plasma samples.		[48]
Improved hMe-Seal	Determining the genome-wide distribution of 5-hmC by selective labeling as enrichment strategy	Using the T4 bacteriophage beta-glucosyltransferase to install a glucose moiety with an azide group onto the hydroxyl group of 5-hmC. then labeled with biotin, thus enables 5-hmC–containing DNA detection, capture, enrichment and sequencing	Detection limit: ~0.004%	Providing acurate and comprehensive capture of 5-hmC–containing DNA fragments, while still providing high selectivity.	Enable us to understand the role(s) of the 5-hmC modification at molecular, cellular and physiological levels.	[49, 50]
Discrimination of Rare EpiAlleles by Melt ( DREAMing )	Semi-limiting dilution and precise melt curve analysis to distinguish and enumerate individual copies of epiallelic species	cfDNA extraction, bisulite conversion, sample dilution, PCR amplification and derivative melt profile analysis. Melt profile shows a secondary melt peak for fully methylated and heterogeneously-methylated epiallele while melt curve of the unmethylated presents only one peak.	Single-CpG-site resolution in fractions: 0.005%	1.Minimal time and cost using a standard qPCR machine and microtiter plate. 2.‘DREAM analysis’ histogram helps easily visualize epigenetic/epiallelic heterogeneity.	1. The sensitivity of the assay determined by the dominant epiallelic methylation density, and epiallelic species not be accurately represented. 2. Relatively low throughput. 3. Not directly provide sequence information.	[51]

Description of Twelve Common Methodologies of ctDNA Analysis. These developed assays and protocols enable excellent accuracy, sensitivity and specificity in the detection of ctDNA and its variation. And those approaches have their own advantages and perspectives. Those approaches include hybrid-capture-based Liquid Biopsy Sequencing (LB-Seq), DNA clutch probes (DCP), integrated digital error suppression (iDES)-enhanced CAPP-Seq, targeted error correction sequencing (TEC-Seq), nanoplasmic biosensor, Simple multiplexed PCR-based barcoding of DNA, Sensitive digital quantification of DNA methylation, Nanostructured conductive polymer platform, tagged-amplicon deep sequencing (TAm-Seq), Single copy sensitive electrochemical assay, Improved hMe-Seal and Discrimination of Rare EpiAlleles by Melt (DREAMing). (AF: allele frequencies)

The detection of ctDNA in background cfDNA released from normal human cells is one of the challenges faced by current ctDNA assays. ctDNA fragments are shorter than plasma background cfDNA fragments, as confirmed in rat brain glioblastoma, rat hepatoma, human melanoma [37], lung cancer [38] and metastatic colorectal carcinoma [9]. Moreover, researches in hepatoma, melanoma and lung cancer [38] also support the increased accumulation of short cfDNA during tumor-related alterations. Thus, the isolation of a set of cfDNA fragments of a specific length by experimental or bioinformatics improvement may increase the detection rate of ctDNA.

Newly developed assays and protocols enable excellent sensitivity and specificity in the detection of ctDNA and its variations, including hybrid-capture-based liquid biopsy sequencing (LB-Seq) [39], which is a strategy that relies on the use of DNA clutch probes (DCP) [40]; integrated digital error suppression (iDES)-enhanced CAPP-Seq [41]; targeted error correction sequencing (TEC-Seq) [19]; nanoplasmic biosensors [42]; SiMSen-seq [43]; methyl-BEAMing [44]; electroactive conducting polymer nanowire platforms [45]; tagged-amplicon deep sequencing (TAm-Seq) [46]; , cMethDNA [47]; and single-copy sensitive electrochemical assays [48]. Methods for determining the genome-wide distribution of 5-hmC (hMe-Seal) [49, 50] and intratumoral epigenetic heterogeneity (DREAMing) [51] by lipid biopsy have also been reported.

Furthermore, using ichorCNA [52], a statistical software, the ctDNA content in cfDNA samples can be assessed to identify if the samples meet the criteria for the full exon sequencing. This method can be used to screen cfDNA samples and identify those that qualify for cfDNA exon sequencing, thus having the potential for use in clinical work. In general, the ultimate aim of ctDNA analysis is to detect early cancers in asymptomatic patients [53]. Despite the aforementioned advantages of cfDNA test methods, their sensitivity and specificity can be undermined by certain challenges. The most striking challenge is the presence of shared mutations between different tumor types, such as KRAS and the EGFR gene, making it difficult to correlate a cancer to a specific organ [53, 54]. However, the exact limitations may vary with the types of tests taken, as listed in the table.

## The caveats and technical issues associated with ctDNA as a biomarker

Since examinations of ctDNA can be performed in a simple and noninvasive manner, this technique has the potential to replace invasive biopsies for patients with insufficient tissue in the first line and progression [55, 56]. A huge advantage of liquid biopsies is the ability to conduct longitudinal monitoring of on-treatment patients as a readout of therapeutic efficacy [57]. ctDNA measurements are stable and have the potential to be used in clinical settings. They can be used as a dynamic biomarker of cancer for early detection, diagnosis and treatment monitoring, and they may also be used to guide patients in choosing adjuvant chemotherapy [58]. However, one of the technical challenges of liquid biopsies is that both healthy and malignant cells release DNA into peripheral blood, thus only a small proportion of cfDNA is tumor-derived ctDNA. In the early stages of cancer, ctDNA levels in cfDNA are lower, making detection more difficult [41, 59]. In addition, ctDNA must be distinguished from cfDNA that is not associated with tumors, especially in patients who have received radiation and chemotherapy [60]. Moreover, the mechanism of ctDNA release and clearance is poorly understood, and the effect of factors such as tumor location is unknown [61, 62] One major concern about liquid biopsies is that mutations of cfDNA in peripheral blood may not be tumor-derived [61]. Therefore, although circulating DNA analysis is promising and convenient, the uniformity of circulating DNA collection and analysis remains insufficient, making the absolute ctDNA amount limited as a diagnostic tool [62].

The purpose of a liquid biopsy is to identify mutations in the target genes and detect emerging resistance to treatment [63]. Therefore, the variation of the ctDNA concentration during treatment appears to be an early biomarker related to therapeutic efficacy. In addition, postoperative ctDNA detection is a marker of residual diseases and a strong indicator predicting the risk of recurrence [64]. In conclusion, the ctDNA level is related to prognosis: a higher ctDNA level is associated with a poorer prognosis. As a result, the ctDNA concentration can be analyzed to change the alternative therapy earlier and minimize side effects [65, 66]. Beyond directing systemic therapy for advanced disease, ctDNA can also provide predictive therapeutic information. Predictive biomarkers can help identify patients who may respond to or develop resistance to specific therapies [67, 68]. However, ctDNA has not been standardized as a biomarker, which means that the results of the ctDNA analysis may not be comparable due to technological differences [69]. To fully incorporate liquid biopsies into clinical practice, there is a glaring need to standardize methods, such as the way blood samples are collected and stored, the technical specifications of the assays, and ctDNA isolation [58, 63, 70, 71].

Despite recent progresses, there are several important technical challenges to the wider use of ctDNA: insufficient knowledge of the tumor microenvironment and the immunologic response to ctDNA release in liquid biopsy samples; diagnostic tools must be further refined to detect small amounts of tumor-derived components in the circulation; the analytical sensitivity of sequencing methods must be increased [72]. In addition, ctDNA analysis is generally limited to fragments of DNA and requires *a priori* knowledge of specific DNA aberrations [60, 69]. With the development of detection technology, a new ctDNA sampling technology is urgently needed to overcome the disadvantages of existing methods, such as high cost, slow speed, low sensitivity, and complexity. (The dimethyl dithiobispropionimidate (DTBP)-based microchannel platform [71] is a good attempt.) Additionally, during the experiment, the sensitivity of the detection method should be reasonably regulated to minimize the probability of a false positive or false negative result [63, 73].

The use of ctDNA as biomarkers in clinical practice should meet the following requirements [72]: high analytical validity, combining established prognostic factors with validated prognostic/predictive biomarkers, and close evaluation of the accuracy, reliability, and reproducibility of a test [68]. Additionally, sufficient clinical validity (which assesses the ability of a test to divide a population into separate groups with significantly different clinical outcomes) and clinical utility (which evaluates whether changes in adjuvant therapy guided by ctDNA have a positive effect on prognosis [74]) must also be met.

In conclusion, the clinical use of ctDNA as a biomarker requires addressing some challenges, including the development of accurate, targeted, and technically reproducible analysis methods, followed by prospective validation in a large cohort of patients [53].

## ctDNA and chromatin modification

Chromatin modifications comprise DNA modifications and histone modifications. At present, ctDNA-related studies have focused more on DNA methylation. DNA methylation is the conversion of the cytosine of the dinucleotide 5' end of CpG islands to 5' methyl cytosine (5 mC) in a DNA sequence catalyzed by DNA methyltransferase (DNMT). Although DNA methylation, as one of the earliest discovered gene modification methods, does not change gene sequence, it can turn off the activity of certain genes. Demethylation, in contrast, has the opposite effect. Of note, some chromatic remodeling factors, including lymphoid-specific helicase (LSH), have been validated to play pivotal roles in the tumor progression and prognosis of multiple cancers, including gliomas [75, 76], lung cancer [77–79] and nasopharyngeal carcinoma [80].

Cells remain normal with high levels of tumor suppressor gene expression. However, if hypermethylation takes place in the CpG islands of tumor suppressor gene promoter regions, which means the tumor suppressor gene is silenced, those cells will break away from the normal cell cycle, thus entering a tumorigenesis process. CpG island hypermethylation in the promoter region of the tumor suppressor gene was first discovered in the retinoblastoma-related Rb gene, and it is regarded as a common phenomenon in several kinds of tumors, such as non-small cell lung cancer (NSCLC). Hypermethylation has also been seen in cell cycle-regulation-related genes (p16INK4a, p15INK4a, p14ARF, RASSF 1A, etc.) and apoptosis-related genes (DAPK, TMS1, etc.), among others. Of them, some genes including p16, p53, and RASSF 1A show a hypermethylation status in various types of tumor cells, while some other genes only maintain hypermethylation in specific tumor cells [81].

Measurements of gene methylation in plasma DNA enable the early detection of primary cancer and metastases to other organs [82]. Plasma DNA SHOX2 and PTGER4 methylation [83] can differentiate lung cancer, nonmalignant lung diseases and the healthy state. GADD45a methylation in prostate cancer plasma is apparently higher than in benign prostate tumor plasma [84]. Other circulating methylation markers include SESN3 [82], WIF1, and NPY [85] for localized colorectal cancer, RAS [86], PTK2, WIF1, and NPY [85] for metastatic colorectal cancer, TAC1 [87] promoter for esophageal adenocarcinoma, RASSF1A [81] for thyroid cancer, SEPT9 [88] for hepatocellular carcinoma, and SHOX2 and SEPT9 [89] for head and neck squamous cell carcinomas.

Specific gene methylation detected in circulating DNA before treatment can be a predictive biomarker for anti-cancer therapy efficacy and disease prognosis. Methylation of 14-3-3 [90] could be a predictor of longer survival for NSCLC patients receiving platinum-based chemotherapy. Promoter methylation of O6-methyl-guanine-methyl-transferase (MGMT) [91] could predict glioblastoma and metastatic colorectal cancer patients’ response to the alkylating agents dacarbazine or temozolomide. The same phenomenon has been reported for methylated TAC1 [87] promoter DNA for esophageal squamous cell carcinoma, SHOX2 and SEPT9 [89] for head and neck squamous cell carcinomas.

In addition to single gene methylation analysis, the detection of methylated gene panel could also be a promising marker for cancer diagnosis, prognosis and disease monitoring. Studies concerning the methylated gene panel have been reported in metastatic breast cancer [92], lung cancer [83], ovarian cancer [93] and primary non-small cell lung cancer [94].

Some researchers have proposed a new analytical framework based on the methylated haplotype load (MHL), a block-level metric, rather than single-CpG methylation levels [95]. The design demonstrated superior sensitivity over methods using single-CpG methylation levels as features, probably because the detection of MHL could help distinguish blocks with various degrees of coordinated methylation despite same average levels of methylation. Although the accuracy was unsatisfactory due to the lack of valid reference methylomes of pure adult cell types, testing of methylation haplotypes can be regarded as a promising strategy for the development of a cancer-specific signature and tissue-of-origin map, thus hopefully facilitating the early detection of a tumor and its primary site.

The 5-hydroxymethylcytosine (5 hmC) plays a distinct epigenetic role [96], and loss of 5-hmC has been correlated with cancer metastasis [75]. Research has also shown that 5-hydroxymethylcytosine signatures in cfDNA developed by genome-wide profiling of 5 hmC can distinguish features of specific cancer types and stages and are superior to conventional protein biomarkers and concordant with 5 hmC biomarkers from tissue biopsies. This approach has been performed in lung cancer, hepatocellular carcinoma, pancreatic cancer, colorectal cancer and gastric cancer [50, 67, 97, 98].

When a known somatic mutated gene has a low frequency, the epigenome, especially the methylome, can sometimes be analyzed instead to determine specific biomarkers for cancers [99]. Moreover, a study in metastatic colorectal cancer demonstrated that the measurement of NGS together with methylated biomarkers in cfDNA could achieve better accuracy than NGS alone [86].

## ctDNA and therapeutic resistance

According to recent studies in different types of cancer, ctDNA is regarded as an applicable, sensitive, and specific biomarker not only for diagnosis but also for monitoring of anti-cancer therapy [100]. Detection of ctDNA variants before and after anti-cancer therapy could provide profound information for therapeutic resistance prediction, evaluation of therapy efficacy and tumor dynamics monitor during treatment, thus facilitating individualized treatment decisions [101, 102].

### Surgery

The level of ctDNA and mutation frequency commonly changes after successful curative surgery. Before surgery, cancer patients have high level of ctDNA. The level generally decreases upon anti-cancer therapy, such as resection, chemotherapy and radiation therapy. If residual disease exists and clinical recurrence occurs, the level would rapidly increase [19]. In addition, ctDNA could assist both quantitative and qualitative assessments of disease progression as a continuous variable correlated with outcome [19].

In patients with resectable colorectal cancer, higher amount of preoperative [19] circulating tumor DNA has been associated with disease recurrence and poor prognosis. However, more researchers have focused on postsurgical plasma samples. Studies in ovarian cancer [103], pancreatic [104, 105] adenocarcinoma, colorectal cancer [74, 106, 107] have indicated that detection of ctDNA after surgery predicted clinical relapse and poor outcome. Furthermore, the results in locally advanced rectal cancer (larc) [74] demonstrate that the detection of circulating tumor DNA prior to any treatment is not predictive of disease recurrence. Postsurgery ctDNA level analysis could stratify cancer patients into subsets that are at high or low risk of relapse, thus aiding the selection of subsequent adjuvant treatments, such as chemotherapy [74, 107]. ctDNA is considered to be superior for detecting relapse than protein tumor biomarkers [19, 106, 108] and CT scan [104, 105] because it has a higher positive predictive value than protein tumor biomarkers and can detect the condition up to 6.5 months earlier than with CT imaging in pancreatic [104, 105] adenocarcinoma.

### Drug

ctDNA has significant advantages in the real-time monitoring of drug efficacy due to its biological properties. ctDNA detection of cancer patients allows the early assessment of the drug response, especially the identification of therapeutic resistance, thus helping physicians improve therapeutic strategies in a timely manner to reduce drug toxicity and achieve better efficacy.[109].

Taking the lung cancer field as an example, by detecting mutations in specific genes in ctDNA, there is a possibility that researchers could predict the following drug response. Patients with detectable EGFR gene mutations in circulating tumor DNA, which is generally concordant with that of tumor tissue [110], may have a higher response rate to epidermal growth factor receptor tyrosine kinase inhibitor (EGFR-TKI) than unmutated patients [111]. Moreover, analysis of circulating tumor DNA can supplement the identification of acquired resistance-associated mutations in patients with advanced cancer.[24] For example, the detection of T790M implies that the patient is likely to develop tolerance to gefitinib, erlotinib, or a combination of erlotinib and pertuzumab. [112] The presence of KRAS mutations in plasma may be a marker of a poor response to chemotherapy. [113] Moreover, monitoring of circulating DNA is informative for earlier evaluation of the treatment response than the radiographic image [114]. If the drug is effective, the drug-sensitive tumor-specific mutations in ctDNA will usually be reduced; otherwise, drug resistance will be manifested in elevated ctDNA levels. Interestingly, after EGFR-TKI therapy, ctDNA with specific mutations also sometimes transiently increases. This phenomenon may be observed in tumors with a few dead cells, for dead tumor cells increase due to drugs and thus release more ctDNA [115].

As occurs in lung cancer, circulating DNA can act as a response, resistance and prognosis biomarker in prostate cancer. BRCA2 reversion mutations are associated with the resistance of inhibitors of the DNA repair protein poly (ADP)-ribose polymerase (PARPi), such as olaparib and talazoparib in prostate cancer patients.[116] Evaluation of the plasma androgen receptor (AR) gene status (including AR amplification, multiple AR mutations, etc.[117]) identifies castration-resistant prostate cancer (CRPC) patients with a worse outcome and resistance to conventional prostate cancer drugs such as enzalutamide and abiraterone [118, 119]. In addition, the detection of RB1 loss [120] and germline DNA repair defects [117] is also associated with the poor response to therapy targeting AR. A study analyzing targeted and whole-exome sequencing of serial circulating-free DNA (cfDNA) samples collected during a Phase II trial (TOPARP-A) of the PARPi olaparib in metastatic prostate cancer demonstrated that cfDNA analyses have the potential to detect all somatic mutations identified in tumor biopsies as well as new mutations emerging only upon disease progression. These new mutations may be caused by therapeutic selective pressures [121].

Also in the breast cancer field, in a phase III clinical trial of breast cancer treatment with CDK4/6 inhibitors, the detection of changes in ctDNA PIK3CA levels after 15 days of treatment predicts progression-free survival (PFS) after treatment [122]. Additionally, analysis of the ESR1 mutation in ctDNA can be used to predict whether patients will be resistant to the next aromatase inhibitor treatment. Although ESR1 mutations are rarely detected in ctDNA in helper aromatase inhibition therapy, they often appear during the treatment of metastatic lesions, suggesting that micrometastases and apparent metastatic cancers have different mechanisms of resistance to targeted therapies [123]. As a whole, early monitoring of ctDNA levels during treatment can predict the efficacy of the therapeutic regimen in patients.

Regarding other types of cancers, research has indicated that multiple recurrent point mutations of ctDNA fibroblast growth factor receptor (FGFR) 2 kinase domain can be detected during the progression of acquired anti-BGJ398 intrahepatic cholangiocarcinoma (ICC). This result indicates that ctDNA FGFR2 mutations could be used to identify resistance of the FGFR inhibitor BGJ398[124]. Studies examining circulating free DNA responses to the drug have also been conducted in ovarian carcinoma [125], colorectal cancer [65, 126, 127] and pancreatic cancer [128]. Moreover, regorafenib seems to be consistently associated with a clinical benefit in patients based on mutational status and protein biomarker concentration, indicating that the detection of circulating DNA could be a viable approach for noninvasive analysis of the tumor genotype in real time [129].

ctDNA can supplement tumor tissue analysis in evaluations of anti-cancer drug responses to help design new strategies for personalized treatment. ctDNA analysis may be more convenient in clinical applications because it can significantly reduce the data turnover time. However, its clinical value still requires support from additional large-scale studies.

### Radiotherapy

Radiotherapy is the mainstay treatment modality for many cancers. However, the increased incidence of recurrence and distant metastasis may lead to the emergence of radioresistance [130]. Detection of circulating DNA may be a criterion for assessing whether postoperative radiotherapy is required. Detection of cfDNA quantification and KRAS and EGFR mutations in the plasma of 168 patients with lung cancer before and after surgery [131] have shown that the analysis of cfDNA could be regarded as a supplement to tissue biopsy. Grading lung cancer patients at cfDNA levels 30 days after surgery may help to select patients who need to undergo chemoradiation after surgery.

## ctDNA and metastasis

The invasion and metastasis of tumor cells are prominent features that are closely related to the prognosis of patients. In clinical practice, the diagnosis of tumors should be performed simultaneously with the diagnosis of primary tumors, lymph node metastasis, and distant metastasis. The tumors are defined as different stages, and the treatment plans are developed according to their stages. At present, the assessment of tumor staging before surgery depends on CT and other imaging methods and biopsy. Recent studies have shown that analyzing the ctDNA of a patient may be helpful in understanding the condition of a patient's metastasis. The level of ctDNA was detected in 640 patients with different types of tumors [100], and the results showed that ctDNA was detected in >75% of advanced patients but <50% of early patients. The analysis of patients with a single kind of tumors also showed that the level of ctDNA might be associated with tumor metastasis. In stage II-IV NSCLC patients, ctDNA can be detected in 100% of the blood sample, and only 50% of patients in stage I can be detected [96]. They also found that the level of ctDNA in newly diagnosed patients with advanced lung cancer is associated with bone metastases and liver metastases [132]. The level of ctDNA in patients with early and late tumors may be related to a variety of factors. In addition to the tumor volume [125, 133, 134], it is also associated with factors such as necrosis, ki67, pathological type, lymph node metastasis, hematogenous metastasis, allele frequency, EpCAM-positive CTC mutation [135] and others [136]. The condition of ctDNA not only reflects the tumor burden but also relates to tumor metabolism, which indicates the biological behavior of the tumor [21].

Interestingly, different metastatic lesions have their own characteristics, and ctDNA detection can reflect these differences [126]. In therapy-resistant patients with colorectal cancer and liver metastases, MEK1 (K57T) resistance mutations are detected by tissue biopsy and ctDNA detection. The ctDNA analysis can also detect previously undetected KRAS (Q61H) mutations [65]. The mutation level increases after treatment, and the mutation is detected in a separate metastatic biopsy. Increased levels of FGFR3 and PIK3CA mutations in blood and urine ctDNA in bladder cancer patients suggest tumor metastasis [137]. A recent study followed the evolutionary perspective of the ctDNA profiles of NSCLC primary tumors and the presence of seven different metastatic lesions. The metastases originated from the same subclone. With the appearance of metastasis at various sites, the ctDNA profile of patients gradually changed. The researchers could observe several mutations in the characteristics of the primary tumor, mutations in the metastatic features, mutations in the primary tumor and metastasis, and mutations in the metastatic characteristics in one site [99]. These results show that the detection of ctDNA may reflect the presence or absence of metastasis in the tumor and offer suggestions about the site of metastasis. It is also worth noting that assessing individual foci in the future may not be sufficient to determine appropriate treatment options.

Monitoring ctDNA during treatment may detect the metastasis as early as possible. A retrospective study comparing current clinical surveillance methods found that in an average of 86% of patients who had passed ctDNA testing, metastasis could be detected 11 months earlier [138].

Moreover, remarkable concordance of driver DNA alterations in ctDNA and matched metastatic tissue biopsies was discovered in mCRPC. This finding suggests that ctDNA assays can be confidently used to molecularly stratify patients and predict prognosis [139]. However, essential drivers of therapy resistance clearly detected in ctDNA can be missed by a single metastatic tissue biopsy. Therefore, an important advantage of ctDNA is its ability to integrate somatic information from more than one metastatic lesion and thereby survey the intrapatient tumor heterogeneity.

## ctDNA and tumor metabolism

Tumor cells have specific types of metabolism to facilitate tumor growth, which is known as the Warburg effect. The common feature of this phenomenon is increased glucose uptake and fermentation of glucose to lactate.[140] Based on the phenomenon that tumor cells take up more glucose than normal cells, in recent years, 18F-fluoro-D-glucose positron emission tomography/computed tomography (18F-FDG PET/CT) has been used to detect tumor metabolic activity.[141] Many studies have shown that 18F-FDG PET/CT facilitates tumor diagnosis, prognosis prediction and assessment of the therapeutic response.[141–145] Moreover, studies examining the correlation between cfDNA and metabolic tumor burden (MTB) measured by 18F-FDG PET/CT have also be conducted. Compared with 18F-FDG PET/CT, the cfDNA level is not a simple measure of tumor burden, but it correlates to a complex tumor metabolism and biology, which makes it a better biomarker to reflect the biological behavior or aggressiveness of the tumor. [21, 146, 147] In addition to 18F-FDG PET/CT, lactate dehydrogenase (LDH), which catalyzes the final step of glycolysis, has also been described by researchers. Serum LDH is also a possible marker for metastasis, prediction of therapeutic efficacy and prognosis.[148–151] Studies on melanoma have demonstrated that ctDNA has a higher sensitivity than LDH to detect disease progression and treatment response, making it a more promising blood-based biomarker than LDH.[152, 153]

Mitochondria provide energy for cell functioning and contain their own genetic material, mitochondrial DNA (mtDNA), which encodes 13 proteins of the mitochondrial proteome. Mitochondrial DNA and protein are of great importance for bioenergetics, especially oxidative phosphorylation, in both normal and tumor cells [154]. Alterations of mtDNA have been observed in multiple kinds of cancers [155]. Moreover, mtDNA damage may be involved in tumor progression and metastasis [156]. As a result, mitochondria are considered to be a possible therapeutic target. For example, mitochondrial proteins, such as Lon protease, Mitofusin-2, and TFAM, among others, may be potential therapeutic targets [157] for bladder cancer. Mitochondrially targeted vitamin E succinate (MitoVES) [158] can influence mtDNA transcripts, inhibit mitochondrial respiration, reduce the generation of reactive oxygen species, and thus suppress the proliferation of cancer cells. A photochemotherapy (PCT) [159] strategy targeting mtDNA using near-infrared (NIR)-assisted tumor-specific Fenton reactions has also been proposed. In vitro and in vivo experiments have also confirmed that inhibition of gasotransmitter hydrogen sulfide (H2S)-producing enzymes increases the sensitivity of lung adenocarcinoma cells to chemotherapeutic agents via induction of mitochondrial dysfunction [160].

Compared with nuclear DNA, mtDNA has a higher copy number per cell and a higher mutation rate, which makes tumor-specific circulating mtDNA a potential biomarker of tumor “liquid biopsy” [161]. Recent studies have shown that the detection of circulating mtDNA content and tumor-specific mtDNA mutations could be a noninvasive tool to predict the risk of developing bladder cancer [157] and hepatocellular carcinoma in HBV patients [7]. mtDNA is also a potential marker in blood for the early detection of bladder cancer [157] and glioblastoma [162]. Elevated mtDNA levels have been observed in the plasma of prostate cancer patients with poor 2-year survival, which indicates that circulating mtDNA could be used to predict prognosis [163].

Although exome sequencing can accurately detect mitochondrial single nucleotide polymorphisms, (SNPs) [164], whether it is feasible to trace mtDNA in blood with current methods remains uncertain. A recent study showed only 17% tumor-specific mtDNA variants were detected in cfDNA with much lower allele frequencies and extensive heterogeneity [161]. Therefore, detecting approach should be improved before further study of circulating mtDNA.

## ctDNA, tumor heterogeneity, and therapeutic resistance

Many studies have confirmed that ctDNA test results are related to drug resistance in cancer patients. Moreover, clinical practice and basic research have validated that the existence of tumor heterogeneity fuels resistance, thus leading to the failure of anticancer therapy. Analysis of ctDNA chromatin modifications can display the heterogeneity of tumors and has great potential for predicting tumor drug resistance.

Tumor heterogeneity refers to the presence of differences in tumor tissue, which mainly contains the following aspects: 1. Individual differences: Patients with the same type of tumor have differences in pathology, tumor biobehavior, responsiveness to treatment, and prognosis. 2. Intratumoral heterogeneity (spatial heterogeneity): In a single patient, there are many differences ranging from genotypes to phenotypes among each tumor cell located in the same tumor tissue block. 3. Temporal heterogeneity: Alterations in the features as well as the genetic makeup of tumors at different stages of development (e.g., primary tumors and metastases) [165].

The presence of tumor heterogeneity, especially intratumoral heterogeneity, leads to the inefficacy of single anti-cancer therapy (targeted drug therapy, in particular) since it is mostly only effective for a portion of the tumor cells. Taking melanoma as an example [166], some patients with melanoma have been clinically found to have poor outcomes when given BRAF/MEF inhibitors, and further studies have found that the tyrosine kinase receptor AXL is highly expressed on the surface of tumor cells that react poorly to BRAF/MEF inhibitors. The sensitivity of tumor cells to AXL-107-MMAE treatment depends on the expression level of AXL receptor on the tumor cell surface, which inspired the synthesis of AXL-107-MMAE, a drug that can effectively kill tumor cells with a high level of AXL via combining AXL antibody with the microtubule-damaging drug monomethyl auristatin E (MMAE). Similar cases have also been reported in myeloma [167, 168], NSCLC [169], breast cancer [170], and colon cancer, among others. Tumor heterogeneity also plays a significant role in acquired drug resistance, the mechanisms of which remain equivocal [171]. To tackle the therapeutic difficulties introduced by tumor heterogeneity, tumor subpopulations should be divided according to the specific characteristics of each subpopulation. For instance, topographic single cell sequencing (TSCS) was utilized to analyze genomic copy number profiles in a tumor subpopulation, and a multiclonal invasion model between ductal carcinoma in situ (DCIS) and invasive ductal carcinoma (IDC) was established [172], which provided insight for tagging tumor subpopulations and their evolution. Accordingly, new drugs targeting those specific markers should be developed for multidrug combination therapy. In contrast, simple and accurate detection methods should be designed to locate and analyze tumor heterogeneity, shedding light on optimized treatment decisions.

The analysis of ctDNA can reflect tumor heterogeneity, hence improving drug resistance prediction and therapeutic decisions. In the study of tumor tissue DNA and paired ctDNA, researchers found that some patients' partial mutation sites could be detected in ctDNA but not in tumor tissues. This phenomenon can be observed in colorectal cancer, myeloma, metastatic castration-resistant prostate cancer and lung cancer [39, 110, 139, 173]. In addition, considering that ctDNA is derived from the passive release of systemic dead tumor cells and the active release of tumor cells, ctDNA is able to paint a better picture of the genomic alterations of tumor patients and eradicate misleading tumor heterogeneity in both tumor diagnosis and drug resistance analyses. However, the source of circulating DNA remains diverse. In addition to tumor cells, circulating DNA can be derived from leukocytes [97] and epithelial cells, among others. Solutions include the establishment of rigorous calling parameters to maintain the platform specificity, potentially at the expense of analytical sensitivity in terms of the detection of low-frequency mutations [19]. As a result, the further application of ctDNA detection in clinical practice requires advanced techniques to reduce the effects of background DNA without compromising its sensitivity.

**Fig. 3:**
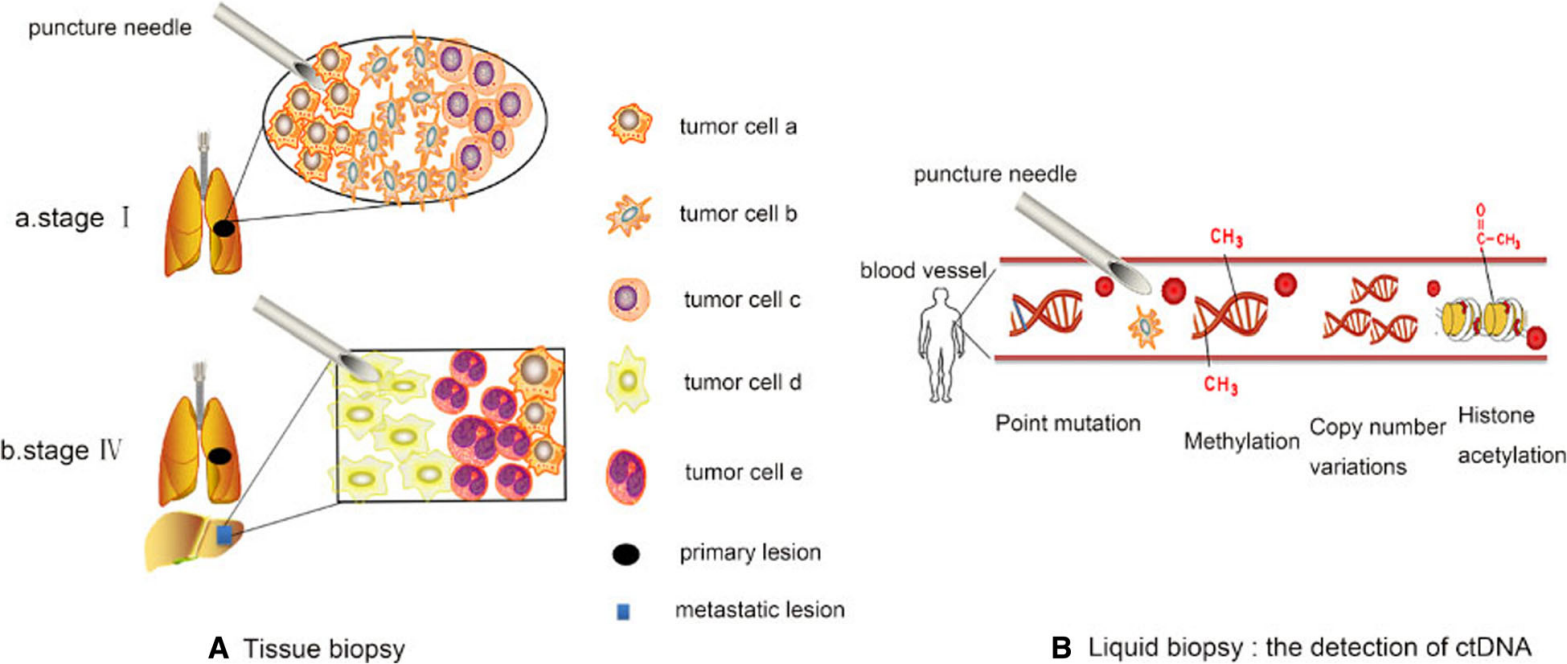
Comparison of traditional tissue biopsy and “liquid biopsy”. **a**: Diagnosis and anti-tumor therapeutic decisions are limited by temporal and intratumoral heterogeneity using traditional tissue biopsies. A mass of tumor tissue consists of several blocks of tumor cell subtypes, while tumors simultaneously at different stages of development (e.g., primary tumors and metastases) can also carry different features. Therefore, samples obtained from tissue biopsy may not provide comprehensive information for diagnosis and therapeutic decisions. **b**: Analysis of ctDNA can present profiles of point mutations, methylation, copy number variations, and histone acetylation, among others, thus providing relatively comprehensive information about the tumor of interest

Compared with tissue biopsies, ctDNA presents a more comprehensive picture of the existence of various subpopulation cells in a tumor, given that plasma samples theoretically contain ctDNA from multiple metastatic examples (Fig. 3). In some scenarios, ctDNA might enable the identification of alterations that were not detected by tissue genotyping, some of which have therapeutic implications. For instance, a clinical study [174] has shown that patients with MET-amplified esophagogastric cancer (EGC) develop resistance after about two months of MET inhibitor therapy, mainly due to the rapid growth of non-MET (EGFR or HER-2) amplified tumor cell subpopulations. Test results of those patients revealed that before and during the use of MET inhibitors, an increase in copy number of EGFR can be observed in ctDNA. Additionally, the increase in ctDNA EGFR copy number is consistent with the growth of MET inhibitor resistant tumor cells. However, the sensitivity of ctDNA platforms had previously limited the application of ctDNA-based analyses in patients with localized and invasive cancers, although developments in the past year have improved the outcome in patients. As a result, researchers have exerted numerous efforts to optimize method selection and invent novel technologies. Taking NSCLC as an example, multiple cross-platform comparisons of technologies encompassing digital and nondigital platforms have been conducted to detect EGFR mutations. Most of the results have demonstrated that both platforms are capable of sensitive and specific detection of EGFR-TKI-sensitizing mutations using patients’ plasma samples [175, 176].

In contrast to current ctDNA detection approaches that typically interrogate a single locus and have low multiplexing capabilities, such as digital droplet PCR60, next-generation sequencing (NGS) methodologies can be used to interrogate larger portions of the tumor genome and track multiple tumor-associated mutations. For instance, some researchers utilized a ctDNA-based NGS analysis of blood samples in 179 patients and identified three subgroups according to their genetic mutations, which favored predictions of drug resistance [177]. Similar practices have also been adopted in prostate carcinoma [178], colorectal cancer [86], and hepatocellular carcinoma [179] among others. Of note, ct-DNA-based NGS analyses have also been conducted in a much larger cohort of 670 patients with more diverse tumors, with encouraging results [180]. In addition, an ultradeep ctDNA-based NGS assay was developed, which underlined the feasibility of performing mutational analysis for 61 cancer-related genes with a simplified workflow, as well as linking cfDNA with systemic treatment success [181]. In summary, the aforementioned advancements will greatly encourage broader investigations of the application of this technology for precision medicine in cancer management.

## Clinical trails

A growing interest in precision medicine and the availability of targeted agents has heightened interest in genomic biomarker-based clinical trials. Trials of targeted therapies intended to show enhanced efficacy in patient subpopulations are increasingly common, such as those with a known biomarker value or genetic tumor mutation. By enabling smaller trials, prognostic enrichment can produce greater efficiency in evaluating new interventions, with potential benefits for patients, sponsors, and public health. With the assumption that preliminary results show evidence that a biomarker such as ctDNA has predictive value, there has been a surge of interest in biomarkers for multiple purposes [182], including the early detection of disease, improved diagnosis and treatment optimization.

There have been 141 clinical trials of ctDNA worldwide, and a total of 17 projects have been completed to date. From the perspective of regional distribution, Europe and East Asia are the main concentrated areas. Among experimental studies of tumors, non-small cell lung cancer has accounted for a total of 42 items, gynecological tumors including breast cancer, ovarian cancer and cervical cancer a total of 20, colorectal cancer 17 items, and prostate cancer and pancreatic tumors 5 items each. There are also a number of clinical studies on tumors such as lymphoma, liver cancer, and head and neck cancer. Based on the type of experimental research, the vast majority of studies are open-label as well as observational studies, and approximately forty percent are parallel assignment. The primary purpose of these experiments is to explore the clinical role of ctDNA as a biomarker for liquid biopsy and patient recruiting. They also focus on the changes in the efficacy of the related chemotherapy and dynamically monitor the efficacy of radiotherapy in combination with imaging. Additionally, one popular use of biomarkers is for the “prognostic enrichment” of clinical trials. There are also institutions that have conducted clinical trials on immunotherapy for current research hotspots. For instance, IMPACT designed by the Shen Lin team at Peking University, is ongoing, the subject of which is identifying MSI status from ctDNA in Chinese patients with refractory advanced solid tumors. The primary outcome measure of the trial is the incidence of MSI-H across different cancer types in Chinese patients at 2 years diagnosed by SPANOM. The estimated enrollment will be 8000 participants, which means the outcome may improve immunotherapy treatment of Chinese patients.

Therefore, we highlight some practical considerations and challenges faced within selected recently completed or ongoing biomarker-based clinical trials investigating ctDNA.

There are few trails with available study results, one of which is NCT02418234. The final results of the NCT02418234 trial and its inherent challenges are as follows: a) the enrollment comprises 314 patients, 56.4% female and 43.6% male, and nearly 80% were stage IV; and b) 97 of the 314 patients with NSCLC resistance to tyrosine kinase inhibitors (TKIs) carried the T790M mutation in ctDNA, as detected by ARMS assay.

An ongoing c-TRAK-TN prospective, multicenter study aims to assess whether ctDNA screening can be used to detect residual disease following standard primary treatment for triple-negative breast cancer, and it will also assess the safety and activity of the investigational medicinal product pembrolizumab. Between 2017 and 2022, over 200 patients aged 16 years or older were recruited in the UK. Blinded serial ctDNA screening every 3 months from the point of registration and completion of primary treatment for triple-negative breast cancer will be performed in the same sample. If a ctDNA positive result occurs on or before the 12-month ctDNA screening assessment, the patient will be randomized by the ICR-CTSU at a 2:1 ratio to the pembrolizumab treatment arm or observation arm. LIBERTI, a prospective, multicenter clinical study in the US aimed to learn more about changes in cell-free tumor DNA in liquid biopsy as they relate to treatment and response to treatment. Another goal of the study was to correlate the presence of ctDNA following complete surgical resection with disease-free survival in more than 500 patients with early-stage NSCLC by evaluating the clinical performance of ctDNA as a signal indicative of the relationship between changes in ctDNA during surveillance and tumor relapse.

Another newly open marker-targeted master protocol is SPECIAL, which uses next-generation sequencing (NGS) and whole-exome sequencing (WES) to assign patients with metastatic solid tumors to individual single-arm targeted studies, in which it is hypothesized that an enhanced tumor response will be achieved with targeted therapy. Additionally, patients will undergo tumor and blood sample collections at serial time points (maximum of 3) to investigate the clonal evolution of tumors under the selective pressure of ICIs. Because a single SPECIAL subprotocol will enroll 14 patients from a number of different tumor types (e.g., breast and colon) that likely have different prognostic or treatment response profiles, it is possible that the primary endpoint (tumor response rate) of the subtrial will vary across organ classes regardless of the marker status and challenge interpretation of the results. The SPECIAL trial was initiated in April 2015 in Canada.

## The drawbacks of ctDNA in clinical applications

Despite the skyrocketing development of ctDNA analysis mentioned above, it is important to accentuate that the efficacy of this model under clinical settings is yet to be determined. To be more specific, current drawbacks hindering its wide-scale clinical applications can be elaborated as the following.

The preanalytical phase mainly contains the identification and selection of an appropriate test, specimen collection and transport. However, several obstacles hinder the proper implementation of the preparation. First, there is a dearth of consistent, reproducible and trustworthy protocols encompassing the blood sample collection, isolation and purification of ctDNA from blood and transport. Without uniform and optimized guidelines for the procedures, poor preanalytical sample handling techniques could weaken the test results, introduce additional costs, or even lead to adverse events in some patients [183]. For instance, there is great discordance between the use of ctDNA collecting tubes, which mainly include ethylenediaminetetraacetic acid (EDTA), CellSave, cell-Free DNA blood collection tubes (BCTs) and Streck. They each cast different effects on cfDNA levels, making them suitable for their respective research purposes [184, 185]. The low isolation efficiency and incompatibility with analytic techniques of ctDNA isolation kits must also be addressed in future studies [186]. In addition, there is an urgent necessity to further explore the effects of the storage temperature as well as the preservation time, as unfavorable conditions may increase cell lysis and interfere with the test results [187, 188]. To effectively circumvent factors that may degrade or contaminate the ctDNA specimen, exhaustive studies are needed to evaluate the influence of the repertoire of variables concerning specimen processing, which contributes to the establishment of an enhanced ctDNA analysis pipeline. Moreover, ctDNA analysis should be a purpose-driven test, with sufficient *a priori* knowledge of mutations of interest (e.g., KRAS, BRAF mutations in colon cancer) under most circumstances. However, the limited scope of test variants may hamper the detection of broader tumor types or novel mutations.

The analytical phase, however, mainly includes the laboratory assay itself. The number of variants identified by genotyping has increased dramatically, creating challenges for the interpretation of the clinically actionable variants and for delivering meaningful data in a timely fashion to guide patient care [58]. The current situation calls for tests such as next-generation sequencing (NGS)-based methods. Nevertheless, each NGS-based method has its own characteristics, which must be weighed by clinicians when carrying out the assay. The common challenges for these approaches, as a result, mainly lie in the discordance of the sequencing data in different assays, with different established parameters. The equivocal range of variant calls can be variable, and therefore rigorous repeats of independent NGS assays or validation of suspected mutation sites are needed [189].

The postanalytical phase mainly consists of data analysis, interpretation of the results and reporting. In this phase, clear outcome interpretations should be expected before the analysis is implemented [58]. However, the major hurdles that hinder cross-assay interpretations comprise the exact origin of the ctDNA, the shedding behavior of the ctDNA, and the concentration of ctDNA. According to previous knowledge, the origin of ctDNA can vary. Apart from malignant tumor cells, it has been reported that benign tumors (e.g., adenomas) harboring overlapping malignant mutations can also release ctDNA in the early stage [190, 191], which may lead to false-positive test results. Nevertheless, it has also been illustrated that benign lesions are unlikely to release significant quantities of ctDNA, making them less amenable for liquid biopsies [100, 192]. Clonal hematopoiesis with somatic mutations, which is often related to aging and confers high relative risks to hematopoietic cancer in the elderly, does not actually translate into many hematopoietic cancers [193, 194]. Therefore, the low absolute risks of clonal hematopoiesis towards cancer can introduce inaccuracies in the test results and unnecessary patient anxiety. In addition, false-negative results can also derive from the following factors. First, little is known about the shedding behavior of different types of cancer cells, as well as patient-specific factors that may influence cfDNA release. These factors include pregnancy, smoking and autoimmune diseases, among others [195]. As a result, the elusive pattern of ctDNA release might lead to the highly variable fraction of ctDNA originating from the tumor cells, ranging from <0.1% to >50% of the total cfDNA [15]. The anatomical barrier of the human body, mainly the blood-brain barrier, can also cause false-negative results. The ctDNA released from tumor cells into the central nervous systems can be impeded by the blood-brain barrier from entering the circulation [100]. Even for the ctDNA successfully released into the circulation, however, there is a great chance that they might be masked by germline DNA resulting from nontumor cells (such as leukocytes). Moreover, it is noteworthy that since ctDNA is usually conceived as the release products when tumor cells undergo apoptosis or necrotic cell death [196], high numbers of resistant tumor cells can escape the detection of ctDNA analysis. Overall, these underlying problems will perplex researchers in analyzing, interpreting and reporting test results.

Despite the vital significance of ensuring the high analytical validity of ctDNA analysis by improving the peri-phases of ctDNA analysis, the clinical utility and validity of ctDNA should be given even greater importance. In clinical scenarios, clinical validity means that the ctDNA test result is associated with whether the patient truly has cancer, while clinical utility refers to the ability of ctDNA to ameliorate the patient outcome as a biomarker [197]. We have presented the studies that support the clinical validity of ctDNA in the above sections, but it remains uncertain whether ctDNA analysis has clinical utility. There is a lack of appropriately designed clinical trials for the validation of ctDNA analysis, whether they be ctDNA stand-alone diagnostic tests or tests that compare information provided by ctDNA with tissue genotyping [195]. Despite some progress [196, 198], further studies are needed to validate the clinical utility of ctDNA.

To conclude, the detection of ctDNA before treatment facilitates the early detection of differential tumor cell subpopulations, thus aiding appropriate treatment decisions. It may also help with the prevention of tumor recurrence by monitoring postsurgical minimal (or molecular) residual disease (MRD) [199]. After the start of treatment, regular monitoring of ctDNA facilitates the early detection of drug resistance due to different mechanisms (acquired genetic alterations or genetic changes that are always present but not detected).

### Perspectives

According to recent advanced studies, ctDNA analysis could be a promising approach to evaluate the treatment response and tumor metastasis condition, thus contributing to the development of the individualized treatment design. More importantly, the analysis of ctDNA is the only method for evaluating liquid biopsies that are recommended by the FDA and the EMA for cancer diagnosis and treatment monitoring. Therefore, ctDNA is a promising biomarker that supplements traditional biomarkers and current screening approaches. However, challenges persist before this technique can be implemented in wide clinical practice in the future. First, further refinement of sequencing is crucial to detect ctDNA specifically with higher sensitivity. Interestingly, recent research has demonstrated improved sensitivity primarily by coisolating the RNA carried in exosomes and ctDNA rather than ctDNA alone, providing an alternative method to maximize the clinical sensitivity of the liquid biopsy assay [200]. Second, research on a larger scale should be conducted using ctDNA sequencing to explore more alterations associated with anti-tumor therapeutic resistance carried by ctDNA. In this case, a better understanding of the ctDNA profile could assist physicians in the treatment design. Finally, more attention could be focused on the exploration of the difference between the primary tumor ctDNA profile and those of metastases to realize the remarkable potential of ctDNA in supervising anti-tumor therapy and evaluating prognosis.

## Abbreviations

5 hmC: 5-hydroxymethylcytosine signatures
AR: Androgen receptor
ARMS: Amplification refractory mutation systems
cfDNA: Circulating free DNA
CRPC: Castration resistant prostate cancer
CTCs: Circulating tumor cells
ctDNA: Circulating tumor DNA
DCIS: Ductal carcinoma in situ
DCP: DNA clutch probes
ddPCR: Droplet digital polymerase chain reaction
DNMT: DNA methyltransferase
DREAMing: Detecting intratumoral epigenetic heterogeneity
DTC: Disseminated tumor cells
EGC: Esophagogastric cancer
EGFR: Epidermal Growth Factor Receptor
EGFR-TKI: Epidermal growth factor receptor tyrosine kinase inhibitor
FGFR: Fibroblast growth factor receptor
ICC: Intrahepatic cholangiocarcinoma
IDC: Invasive ductal carcinoma
iDES: Integrated digital error suppression
LB-Seq: Liquid biopsy sequencing
mCRPC: Metastatic castration-resistant prostate cancer
MGMT: Methyl-guanine-methyl-transferase
MHL: Methylated haplotype load
MRD: Minimal residual disease
NGS: Next-generation sequencing
NSCLC: Non-small cell lung cancer
PARPi: Poly (ADP-ribose) polymerase inhibitor
PFS: Progression-free survival
TAm-Seq: Tagged-amplicon deep sequencing
TEC-Seq: Targeted error correction sequencing
TSCS: Topographic single cell sequencing
